# Establishing a normative database for quantitative pupillometry in the pediatric population

**DOI:** 10.1186/s12886-020-01389-x

**Published:** 2020-03-26

**Authors:** Sanket S. Shah, Hantamalala Ralay Ranaivo, Rebecca B. Mets-Halgrimson, Karen Rychlik, Sudhi P. Kurup

**Affiliations:** 1 Department of Ophthalmology, Northwestern University Feinberg School of Medicine, Chicago, IL USA; 2 Division of Ophthalmology, Ann & Robert H. Lurie Children’s Hospital of Chicago, Chicago, IL USA; 3 Stanley Manne Children’s Research Institute, Chicago, IL USA

**Keywords:** Pupillometry, Pupils, Pediatric screening, Normative

## Abstract

**Background:**

Pupillary evaluation is a crucial element of physical exams. Noting size, reactivity, and consensual response is critical in assessing for optic nerve dysfunction. We aim to establish normative data for scotopic pupillary size and function in the pediatric population in a clinical setting.

**Methods:**

Pupillometry was obtained prospectively for consecutive, normal patients < 18 years old being evaluated by Lurie Children’s Ophthalmology. Quantitative data included maximum (MAX) and minimum (MIN) diameters, constriction percentage (CON), latency (LAT), average (ACV) and maximum (MCV) constriction velocities, average dilation velocity (ADV), and 75% recovery time (T75). Iris color was noted as light, intermediate, or dark.

**Results:**

196 eyes of 101 participants (42.6% male, ages 1–17 years, average age 10.3 years) were analyzed. Mean MAX was 6.6 mm (5.1–8.1 mm 95% CI); MIN was 4.7 mm (3.1–6.1 mm 95% CI); CON was 30% (17–42 95% CI); LAT was 230 milliseconds (160–300 ms 95% CI); ACV was 3.70 mm/sec (2.21–5.18 mm/sec 95% CI); and ADV was 0.88 mm/sec (0.38–1.38 mm/sec 95% CI). Age had a positive correlation with MAX, MIN, and CON. 84.2 and 95.8% of participants showed resting pupil asymmetry of ≤0.5 mm and ≤ 1.0 mm, respectively.

**Conclusions:**

Quantitative pupillometry can be a useful tool for screening pediatric patients. We sought to establish normative data in this group. We found males to have significantly greater MCV and CON than females (*p* < 0.05). Also, age had a positive correlation with MAX, MIN, and CON.

## Background

The pupillary exam is a crucial element of ophthalmologic and neurologic evaluations. In particular, observation of pupillary size, reactivity, and consensual response is critical in the assessment of optic nerve dysfunction. In the early 1990’s, some studies showed pupillometry as a more sensitive method to measure autonomic nerve function in adolescents with type 1 diabetes compared to cardiovascular reflexes [1–3]. More recently, quantitative pupillometry has been used to evaluate early changes in intracranial pressure in patients with head injury or neurological disorders [4, 5]. It has also permitted researchers to detect deficits in patients with pre-perimetric glaucoma and diabetes without retinopathy [6, 7]. Furthermore, Kardon et al. showed that chromatic pupillometry could be used by clinicians as a non-invasive method of monitoring retinal functional status, especially in patients with severe retinitis pigmentosa [8]. Quantitative pupillometry has been shown in the intensive care unit to be more accurate, consistent, sensitive, and specific with less interrater disagreement than manual measurement by nurses experienced in neurological examinations [9]. Furthermore, Couret et al. showed that trained nurses in Neuro Critical Care Units did not detect 50% of anisocoria and concluded that automated quantitative pupillometry is a more reliable method with which to collect pupillary measurements at the bedside [10]. Several studies have described normative values [4, 5, 11] for pupillary function in a pediatric population as measured in ambient light conditions. Kohnen et al. published a normative study [12] with scotopic pupil size. This suggests a need for normative values, measured in a clinical setting, in a pediatric population that can be used not only for evaluating patients when suspicious of disease, but also as a more accurate and sensitive screening tool. A normative database with which to compare measured patient quantitative pupillometry values can permit providers of all levels to easily examine and quickly refer patients to eye care providers in necessary situations. The present study aims to establish a normative database for scotopic pupillary size and function in children as measured in a clinical setting.

## Methods

This study was approved by the Institutional Review Board of Ann & Robert H. Lurie Children’s Hospital of Chicago (Lurie Children’s) and conformed to the requirements of the United States Health Insurance Portability and Privacy Act with adherence to the tenets of the Declaration of Helsinki.

Based on the lead author’s availability, pupillometry and informed consent were obtained in a prospective manner for consecutive normal patients younger than 18 years old (i.e. patients without pathology anticipated to affect pupillary function who also meet inclusion criteria) who were being evaluated at the Ophthalmology Clinic of Lurie Children’s. Exclusion criteria for controls included: inability to sit for testing, developmental delay, genetic syndromes, neurologic pathology (including intracranial masses), or intraocular pathology that would affect pupillary function (e.g. uveitis, cataracts, diabetes, glaucoma, optic nerve dysfunction).

Among the 143 patients consented, 42 patients were excluded due to inability to cooperate with the exam (34), inability to perform the exam due practicalities of clinic work flow after consenting [7], or pathology found on exam [1].

Pupillometry data were measured using the handheld NeurOptics PLR-200TM Pupillometer (NeurOptics, Irvine, CA, USA). The pupillometer employs an 850 nm infrared illumination to collect measurements on the eye before, during, and after presentation of a full-field white light stimulus with peak wavelengths comprised of red, green, and blue. An opaque rubber interface that can be sanitized places the device in contact with the orbital rim of the eye to be measured, thus occluding outside light from entering the measured eye and allowing direct measurement. The device highlights an outline of the pupil and graphs its displacement over time with accuracy of 0.05 mm. Data were obtained in a dark clinic room throughout regular clinic hours (8:00 AM – 5:00 PM) to emulate routine clinical testing. Scotopic conditions were verified prior to exam by measurement of luminance of less than 2 Lumens with a luminometer (Dr.Meter® LX1330B Digital Illuminance/Light Meter, Hisgadget, Union City, CA, USA) at the level of the patient’s eyes. The right eye was measured first in each patient. Data from each eye exam were recorded prior to examination of the second eye. Recorded data included maximum (MAX) and minimum (MIN) diameters, constriction percentage (CON) — the calculated percentage difference between MAX and MIN, latency (LAT), average (ACV) and maximum (MCV) constriction velocities, average dilation velocity (ADV), and 75% recovery time (T75).

Iris color was noted as light (blue), dark (brown) or intermediate. Study data were managed using REDCap electronic data capture tools hosted at Northwestern University [13].

### Statistical analysis

Categorical variables are reported as frequencies, and percentages and continuous variables are reported in means and standard deviations. Pearson correlations were used to describe relationships between two continuous variables: age and eye measurements. To account for the random effect of two-eye measurements per patients, mixed effect models were run to determine differences between independent variables including eye color, race, ethnicity, and gender. All tests were two-sided, and significance was defined as *p* < 0.05. Statistical analyses were conducted using SAS version 9.4 (SAS Institute, Inc., Cary, NC).

## Results

Data from 196 eyes of 101 participants were analyzed with the exception of average dilation velocity and 75% recovery time, which could not be measured in 62 and 94 eyes, respectively (usable *n* = 134 and 102, respectively). The self-identified demographic distribution of the population is shown in Table 1.

**Table 1:** Demographics of the pediatric population measured. Race and ethnicity were self-reported by the guardians of each patient

Age (years)	10.33 ± 3.83 (mean ± SD)
Age range (years)	1.01–17.44
Gender	
Male	42.6%
Female	57.4%
Eye Color	
Blue	9.7%
Brown	78.1%
In Between	12.2%
Race	
White	31.7%
African American	7.9%
Asian	8.9%
Other	51.5%
Ethnicity	
Hispanic	42.6%
Non-Hispanic	57.4%

In our study population, the mean maximum resting pupil diameter was 6.6 ± 0.74 mm, and the mean minimal post-stimulation diameter was 4.7 ± 0.77 mm. The mean percentage of reduction in pupil size after stimulation was 30 ± 6.2%. The average latency time was 230 ± 34 ms. The mean constriction velocity was 3.70 ± 0.744 mm/s while the mean dilation velocity was 0.88 ± 0.25 mm/s.

Table 2 highlights the mean pupillometry values for across the entire population we tested and the subsets based on gender. The most commonly encountered iris color was dark (*n* = 133 out of 196 eyes). Males had significantly greater constriction percentage and maximum constriction velocity than females (*p* < 0.05). There were no statistically significant differences between the parameters measured in Hispanic and non-Hispanic subjects (self-reported as ethnicity by all subjects). Race (i.e. White, African-American, Asian, other) was self-reported for 49/101 subjects, and there were no statistically significant differences shown between the parameters measured.

**Table 2:** Quantitative pupillometry values measured under scotopic conditions during this study, reported as mean values of the population and between gender and ethnicity

		Gender		Ethnicity	
Variable	All Children	Male	Female	Hispanic/Latino	Non-Hispanic/Latino
Maximum Diameter (mm)					
Mean (n)	6.6 (196)	6.7 (83)	6.5 (113)	6.5 (83)	6.7 (113)
(Mean - 2 SD, Mean + 2 SD)	(5.1, 8.1)	(5.1, 8.3)	(5.2, 7.9)	(5.2, 7.8)	(5.1, 8.2)
Minimum Diameter (mm)					
Mean (n)	4.7 (196)	4.6 (83)	4.7 (113)	4.6 (83)	4.7 (113)
(Mean - 2 SD, Mean + 2 SD)	(3.1, 6.2)	(3.1, 6.2)	(3.2, 6.2)	(3.2, 6.0)	(3.1, 6.3)
Constriction Percentage					
Mean (n)	−30 (196)	− 31* (83)	−28* (113)	−29 (83)	− 30 (113)
(Mean - 2 SD, Mean + 2 SD)	(−42, −17)	(− 41, − 21)	(− 42, −15)	(− 42, −16)	(− 42, −18)
Latency (ms)					
Mean (n)	230 (196)	230 (83)	230 (113)	230 (83)	230 (113)
(Mean - 2 SD, Mean + 2 SD)	(160, 300)	(170, 290)	(170, 290)	(170, 290)	(170, 290)
Average Constriction Velocity (mm/s)					
Mean (n)	−3.70 (196)	−3.83 (83)	− 3.60 (113)	−3.69 (83)	− 3.69 (113)
(Mean - 2 SD, Mean + 2 SD)	(−5.18, −2.21)	(− 5.17, − 2.49)	(−5.16, − 2.04)	(−5.15, − 2.23)	(− 5.21, − 2.17)
Maximum Constriction Velocity (mm/s)					
Mean (n)	− 5.02 (196)	− 5.28* (83)	−4.83* (113)	−4.91 (83)	− 5.09 (113)
(Mean - 2 SD, Mean + 2 SD)	(−6.81, − 3.23)	(−6.76, − 3.80)	(− 6.73, − 2.93)	(− 6.79, − 3.03)	(−6.83, − 3.35)
Average Dilation Velocity (mm/s)					
Mean (n)	0.88 (134)	0.91 (59)	0.86 (75)	0.87 (55)	0.90 (79)
(Mean - 2 SD, Mean + 2 SD)	(0.38, 1.38)	(0.39, 1.43)	(0.38, 1.34)	(0.37, 1.37)	(0.44, 1.36)
75% Recovery Time (s)					
Mean (n)	2.82 (102)	2.89 (45)	2.77 (57)	2.85 (47)	2.80 (55)
(Mean - 2 SD, Mean + 2 SD)	(1.51, 4.13)	(1.77, 4.01)	(1.33, 4.21)	(1.69, 4.01)	(1.36, 4.24)
Asymmetry					
≤ 0.5 mm	84.2%				
≤ 1.0 mm	95.8%				

*n* Number of eyes. *p < 0.05

There was a positive correlation with age and maximum diameter (*r* = 0.26 *p* = 0.0002), minimum diameter (*r* = 0.28 *p* < 0.0001), and constriction percentage (*r* = 0.23 *p* = 0.0015). Without quantitative pupillometry, anisocoria has been defined as asymmetry of 1 mm or greater [4, 5]. Moreover, pupil asymmetry measuring less than 0.5 mm is difficult to detect with the naked eye. In this study, 95 participants recorded measurements from both eyes. Of these, 84.2% (80/95) and 95.8% (91/95) of participants showed resting pupil asymmetry between 0 and 0.5 mm and 0–1.0 mm, respectively.

## Discussion

Previous studies have evaluated pupillary size in both ambient photopic [4, 5, 11, 14, 15] and scotopic conditions [12, 14, 15]. There has also been assessment of pupillary function in ambient light conditions alone [4, 5, 11]. Table 3 shows the results of pupillary size and function from the aforementioned studies. This study is the first to propose normative values for parameters of scotopic pupillary function in a pediatric population in typical clinical conditions.

**Table 3:** Quantitative pupillometry data reported by previous studies presented in comparison with this study

Study	n	Ages (years)	Light conditions	MAX (mm)	MIN (mm)	CON (%)	LAT (ms)	ACV (mm/s)	MCV (mm/s)	ADV (mm/s)	T75 (s)	Asymmetry
This paper	196 eyes (101 patients)	1–18	Scotopic	6.6 ± 0.08	4.7 ± 0.8	30% ± 6%	230 ± 35	3.70 ± 0.75	−5.02 ± 0.90	0.88 ± 0.25	2.82 ± 0.66	15.8% ≥ 0.5 mm
Taylor et al. [5]	2432 paired measurements (310 patients)	1–87	Ambient light	4.1 ± 0.34	2.7 ± 0.21	34%	240 ± 40	1.48 ± 0.33				< 1% > 0.5 mm
Boev et al. [4]	90 eyes (90 patients)	0–18	Ambient light	4.11	2.65	36%		2.34		2.2		“Normal” < 0.5 mm
Kohnen et al. [12]	166 eyes (83 patients)	6.01 ± 4.11	2 min dark adaptation	6.09 ± 0.98								9.64% ≥ 0.5 mm
Brown et al. [11]	201 eyes	1–18		5.36 ± 0.90	3.62 ± 0.65				4.92 (W) 4.42 (AA)			
Fan et al. [14]	25 patients	18–22	Photopic and scotopic	5.93 ± 0.75 (M,P) 5.53 ± 0.57 (F, P) 6.72 ± 0.86 (M, S) 6.42 ± 0.68 (F, S)								
Tekin et al. [15]	20 eyes (20 patients) 25 eyes (25 patients)	0–10 11–20	Scotopic	2.4 ± 1.1 2.4 ± 1.1		55 ± 7% 57 ± 6%	262 ± 37 242 ± 48	6.1 ± 0.6 6.2 ± 1.0		2.4 ± 1.1 2.4 ± 1.0		

*n* Number of measurements, *MAX* Maximum diameter, *MIN* Minimum diameter, *CON* Constriction percentage, *LAT* Latency, *ACV* Average constriction velocity, *MCV* Maximum constriction velocity, *ADV* Average dilation velocity, *T75* 75% recovery time; (*M,P*) Male, photopic, *F,P* Female, photopic, *M,S* Male scotopic, *F,S* Female, scotopic, *W* White, *AA*, African-american

It is well established that, throughout childhood, the eye and visual pathway have significant growth and development, which may expectantly lead to pupillary changes over time. Although under ambient light conditions, Brown et al. measured data similar to our results that suggested a weak, yet statistically significant, correlation between pupil size and age (resting diameter, *r* = 0.29; post-stimulus aperture, *r* = 0.19). Other studies in light [11] and dark conditions [12] suggest a peak in pupil size around 11 years old, which was shown in light to continue to decrease throughout adulthood [16]. However, we did not see a similar peak and subsequent decrease in our scotopic pediatric data. Our contrast in findings compared to Kohnen et al. might be attributable to differences in population sampling; ours included a larger number of subjects, especially in ages above 11 years old.

In collecting pupillometry data, the device used reports data in two colors: green (reliable) and red (unreliable). It will only display as green if it generates reliable data in at least six out of the eight variables measured. This explains the discrepancies in n values of ADV and T75 from the remainder of the data. Adhikari et al. showed that pupil diameter measured by an infrared camera is affected by fixation eccentricity, introducing at least a 0.07-mm error once eye movements deviate more than 5° from the central fixation axis [17]. While fixation eccentricity was not measured in this study, the authors felt the levels of reported reliability from the pupillometer served as an appropriate surrogate when considering the nature of examining children in a clinical setting. Multiple studies [4, 11, 12] have mentioned the inherent difficulties in measuring children younger than 5 years old. We found this to be true for practical purposes, as well; 28/42 of the patients unable to cooperate with the exam were less than 5 years old. Among these children, the most encountered difficulties were fear of the pupillometer, inconsolable crying, and constant head movement. The remaining 14/42 patients were between 5 and 10 years old. These children cooperated well with the exam, but they were unable to avoid blinking during the pupillary constriction period of measurement — thus preventing adequate data collection. With more experience, younger children were able to be distracted for sufficient periods of time to collect data, and older children tolerated assistance from the examiner’s hand to prevent eye blinking. Thus, proper training and repeated use, just as with other instruments used in eye examination, can yield more quantitative measurements that were otherwise subjectively based. Although we presented a smaller sample size for younger individuals, we hope to provide some amount of normative comparison in that age group. Finally, for future study, a larger sample size can strengthen the significance of a normative database.

Previous studies have been equivocal on the association between gender and pupillary reaction, showing weak differences [14] or no difference at all [11]. Our data show statistical differences only between the constriction percentage and maximum constriction velocities of young males and females (*p* = 0.0179 and *p* = 0.0105, respectively). In contrast to Fan et al., our findings might be attributable to differences in population sampling: ours having a higher n and lower ages than their population [14]. Our data suggest that a potentially abnormal patient should be compared to one of the same gender from our data.

Via self-reporting, demographic data was obtained for all subjects on ethnicity but for only 49/101 subjects with regards to race. There were no statistical differences for any measured variable by ethnicity or by race. However, Brown et al. measured a population that was 64% white and 24% African American, finding statistical differences in pupil size and function (MCV and ADV) between the eyes of White and African American subjects when measure in ambient light. Due to differences in demographics between the two studies, direct comparisons cannot be made.

Anisocoria has been clinically defined as asymmetry of 1 mm or greater [4, 5]. Interstimulus interval between eyes has been shown to affect measurement of pupillary light response amplitudes if the pupil has not returned to its resting diameter, and the necessary interval is shorter in scotopic conditions [18]. Order of eye measurement, thus, could affect anisocoria data if the interstimulus interval is not appropriate. During this study’s data collection, the interval between eye measurements was roughly 1 min while the previous eye data was transcribed. There was no significant trend found in the data to favor the resting diameter of one eye to be greater than the other. As shown in Table 3, multiple studies including the present one show the majority of patients to have pupillary asymmetry of less than 0.5 mm on quantitative pupillometry. Therefore, the authors recommend future investigation into detection of pathology by using thresholds of 0.5 mm and 1 mm for anisocoria to determine the potential utility of quantitative pupillometry over standard subjective measurement as a screening tool as well as determining exact sensitivity, specificity, and receiver-operator curves for each method using these thresholds.

The clinical nature of this study provides both strengths and limitations to the data and analysis presented above. As mentioned earlier, fixation eccentricity and exact interstimulus interval provide a more standardized approach to data collection. Furthermore, data were collected throughout the clinic day, thus preventing consistent time measurements to limit effects from circadian rhythm on pupillary function [19]. Finally, given the clinical setting of these measurements, children are likely to have many psychosensory influences on pupil size and function such as anxiety, fright, and pain, all of which would exhibit mydriatic effects on the data. However, the authors wish to highlight that when clinically evaluating a pediatric population, precise time of day and measurements that require cooperation as well as the psychosensory effect of visiting any clinical setting whether it be in a hospital-based clinic or an outpatient clinic are rarely standard. Therefore, the data presented in this study provide a robust database with which comparisons can be made with a pediatric population in a clinical setting.

## Conclusion

In conclusion, the authors offer quantitative pupillometry measurements in children to be compared against the gender-matched data presented in this manuscript. Children falling outside of normal ranges may warrant further evaluation.

## Data Availability

The datasets generated and/or analysed during the current study are not publicly available because the participants did not consent to have their data available for future research.

## Abbreviations

ACV: Average constriction velocity
ADV: Average dilation velocity
CON: Constriction percentage
LAT: Latency
MAX: Maximum diameter
MCV: Maximum constriction velocity
MIN: Minimum diameter
NCCU: Neuro-critical care unit
T75: 75% recovery time
